# The influence of political ideology and trust on willingness to vaccinate

**DOI:** 10.1371/journal.pone.0191728

**Published:** 2018-01-25

**Authors:** Bert Baumgaertner, Juliet E. Carlisle, Florian Justwan

**Affiliations:** Department of Politics and Philosophy, University of Idaho, Moscow, Idaho, United States of America; Fordham University, UNITED STATES

## Abstract

In light of the increasing refusal of some parents to vaccinate children, public health strategies have focused on increasing knowledge and awareness based on a “knowledge-deficit” approach. However, decisions about vaccination are based on more than mere knowledge of risks, costs, and benefits. Individual decision making about vaccinating involves many other factors including those related to emotion, culture, religion, and socio-political context. In this paper, we use a nationally representative internet survey in the U.S. to investigate socio-political characteristics to assess attitudes about vaccination. In particular, we consider how political ideology and trust affect opinions about vaccinations for flu, pertussis, and measles. Our findings demonstrate that ideology has a direct effect on vaccine attitudes. In particular, conservative respondents are less likely to express pro-vaccination beliefs than other individuals. Furthermore, ideology also has an indirect effect on immunization propensity. The ideology variable predicts an indicator capturing trust in government medical experts, which in turn helps to explain individual-level variation with regards to attitudes about vaccine choice.

## Introduction

One of the most successful public health interventions has been infant and childhood immunization programs. In 1900, 16 out of every one hundred American children died from disease before age five [1]. By the close of the century, 97% of American schoolchildren received vaccines against diphtheria, tetanus, pertussis, polio, measles, mumps, rubella, and Haemophilus influenzae type b (Hib) by first grade [1]. While widespread vaccinations have nearly eradicated what were once very common and deadly diseases, the unfortunate irony is that without threat of such deadly diseases the proportion of the population that is not adequately vaccinated has grown [2]. In 2015, the national vaccination coverage among children aged 19–35 months was 91.9% for recommended MMR (measles, mumps, rubella) doses, but in states such as Colorado, Ohio, and West Virginia the coverage is as low as 86.0% [3]. For measles, the proportion of the population that should be vaccinated for prevention of disease outbreak is 90–95% [4]. Thus, the fact that vaccination coverage is dropping is particularly concerning especially in light of recent outbreaks.

Despite all 50 states requiring children to be vaccinated before attending school, all states allow exemptions for medical reasons, all but two allow exemptions for religious reasons, and almost half allow exemptions for philosophical reasons [5]. Such exemptions contradict the efforts of the U.S. government to adhere to a federally mandated vaccine schedule and achievement of universal vaccination to maintain herd immunity. Various reasons for parents not vaccinating their children exist, from mere oversight [6], socio-economic barriers (that often interact with race/ethnicity) [7], and for some the result of conscious decisions. Oftentimes the deliberate decisions of parents are based on parental concern regarding vaccine safety [8] and efficacy [9,10].

For example, there is a growing parental and public interest in natural products and even some have taken up the mantel to “green our vaccines” due to public fears of the relationship between MMR vaccine and autism (a relationship for which no credible empirical evidence has been found [11]). When vaccinations concern children, as in the case of MMR, parents lack control over the outcome of vaccination and the potential damage, although extremely rare (less than 1 in a million), can be long-term or even fatal [12]. Moreover, benefits can be difficult to calculate, particularly given that the negative consequences of nearly-eradicated diseases are no longer salient. Consequently, many parents give greater weight to the risks of vaccines than the benefits [13]. With the ubiquity of the internet and information available online along with a shifting parent-doctor relationship, parents have become more involved in vaccination decisions and often override the mandated vaccine schedule.

In light of the increasing refusal of some parents to vaccinate children, public health strategies deploy a “knowledge-” or “information-deficit” approach that educates people on the risks, costs, and benefits of vaccination (and non-vaccination). If individuals respond to risk information in a straightforward way, it is reasonable that a knowledge-deficit approach would be successful. However, research across numerous domains suggests that, in general, decision making under risk is complex and not straightforward [14–22]. There is reason to believe that vaccine choice is no different [23]. For example, it is known that perceived risk of vaccines is related to gender (with women perceiving greater vaccine risk than men) as well as a variety of other demographic characteristics such as age, race, education, and income level, and other factors such as emotion, culture, religion, and socio-political context [24]. In this paper, we are interested in two sets of reasons for vaccination decision making and the relationship between them: ideology and trust.

From a sociological perspective, a number of existing empirical studies indicate that people’s ideologies and worldviews strongly influence their perception and acceptance of risk. That is, rather than understanding risk as a result of individual cognition, Cultural Theory, attributable to Douglas [25,26] and Douglas and Wildavsky [27], posits that individuals are embedded in a sociocultural milieu wherein and by which risk is constructed and interpreted [27]. Specifically, Douglas and Wildavsky use four categories—hierarchical, individualist, fatalist, egalitarian—to understand how each cultural group applies salient values and interprets a particular phenomenon to be risky or not. Others have built on their work, substantiating the relationship between these four categories and perception of risk [28–32].

Related, political ideology, defined as the set of beliefs about the proper order of society [33], has a strong influence on political attitudes and behaviors and general value orientations—and by extension risk—in a pattern similar to what scholars find with Cultural Theory. Indeed, some scholars [34] find that some individuals actually respond to egalitarianism and individualism questions as if they were opposite ends of a single, liberal-conservative continuum, rather than two of four distinct worldviews. This finding demonstrates the close correspondence between Cultural Theory and political ideology and the explanatory value of political ideology and risk.

Research further bears out the close correspondence between Cultural Theory and political ideology. Studies find that liberals are more egalitarian and open to change than conservatives [35–43]. Others find political conservatives to be more sensitive to threat and more risk averse than those who are politically liberal [44–46]. Moreover, ideological dispositions can shape support for or opposition to potentially risky technologies [47,48]. In particular, Rothman and Lichter [47] find that ideology is related to assessments of nuclear power safety for some groups including journalists and high-level government bureaucrats. Duckit and Sibley [49] distinguish between social and cultural conservatives (or right-wing authoritarians), who tend to perceive the world as “dangerous” or unstable, and economic conservatives (or those with high social dominance orientation), who perceive “the world as a ruthlessly competitive jungle in which the strong win and the weak lose” [50]. However, both types of conservatives have a desire to reduce uncertainty and threat and “prioritize traditionalism, rule-following, and acceptance of inequality” [51]. Thus previous research provides a conceptual linkage between ideology and risk.

In light of the above research findings, political ideology may be of particular importance in the case of vaccine attitudes. Some might suggest that because vaccinations have not yet been adopted by a major political party or ideological camp (compared to other issues such as abortion), the public should not possess well-developed partisan or ideological opinions about them. Although we concur with this assessment, we also suspect there to be ideological opinions about vaccines in the U.S. as a consequence of the aforementioned relationship that exists between political values and risk (or risky technologies). Moreover, we suspect that conservatives will be less likely to express pro-vaccination attitudes, despite the notorious vaccine skepticism that some liberals, such as Robert F. Kennedy Jr., have adopted. In fact, despite anecdotes attributing anti-vaccination trends to some enclaves of liberal leaning types, evidence points to more vaccination skepticism among conservatives [52]. Additionally, anti-vaccination opinions have been publicly discussed among conservative leaders. For example, during the 2016 presidential race, several Republican candidates expressed some degree of skepticism concerning vaccination [53]. Furthermore, Donald Trump has used Twitter to perpetuate a long debunked linkage between autism and vaccines since as far back as March 2012 [54,55]. By bringing the issue into the highly salient presidential election, Trump could have motivated an ideological gap in public attitudes about vaccination. The above considerations allow us to formulate our first testable hypothesis:

**Hypothesis 1**: Individuals who are more conservative are less likely to vaccinate against preventable diseases than less conservative individuals.

The other set of reasons for anti-vaccination attitudes we are interested in concerns trust, which has been an area of interest for researchers studying vaccination propensity [56]. Trust is particularly important when dissenting opinions exist regarding scientific facts and individuals have to choose between them. For example, are vaccines safe or is there a substantial risk of illness or death? Typically, individuals are unable to answer this question for themselves given that they lack the expertise to test vaccine safety or gather data on vaccination risk. Thus, people need to turn to experts who have either done the research or have access to the relevant information. In this context, we distinguish between two kinds of medical experts: government medical experts and primary health care providers. Anti-vaccination attitudes are often correlated with low levels of trust in the government [9], and lack of trust in corporations and public health agencies [9]. The less people trust governmental or scientific institutions the more likely they are to believe a link between vaccines and autism and thus, the less likely they are to demonstrate support for vaccinations. Given these considerations, we have the following hypothesis:

**Hypothesis 2**: Individuals with high levels of trust in government medical experts are more likely to express pro-vaccination attitudes against preventable diseases than individuals with low levels of trust.

Separate from trust in medical institutions, individual members of the health care community are also likely to influence vaccination attitudes. In particular, high levels of trust in a primary health care provider, e.g., a pediatrician, is expected to result in more positive attitudes towards vaccination than low levels of trust:

**Hypothesis 3**: Individuals with high levels of trust in their primary health care provider are more likely to express willingness to vaccinate against preventable diseases than individuals with low levels of trust.

So far, we have argued that ideology and trust influence vaccine attitudes in individuals. However, there are reasons to believe that these two independent variables also influence each other. As we discussed in our motivation of hypothesis 1, individuals who are more conservative are more likely to be skeptical about vaccination. This may be part of a more general pattern of skepticism towards different types of expertise. We know generally that trust in government vaccination programs, trust in science, and trust in government is usually lower for conservatives than for liberals [57–59]. Thus it is reasonable to expect that there are lower levels of trust towards our two types of medical experts among more conservative individuals than less conservative individuals:

**Hypothesis 4**: Individuals who are more conservative are less likely to trust government medical experts than less conservative individuals.
**Hypothesis 5**: Individuals who are more conservative are less likely to trust primary health care providers than less conservative individuals

We have additional reasons for expecting support for hypothesis 4, that individuals that are more conservative are less likely to trust government medical experts than less conservative individuals. There is evidence that trust in government medical experts, such as the Centers for Disease Control and Prevention, can be affected by ideological triggering. This can happen by signaling group identity, e.g., through partisan news outlets. Receiving information through partisan outlets allows individuals to selectively credit information related to vaccine risks and benefits in ways that reflect their ideological dispositions (e.g., vaccines against sexually-transmitted disease would lead to an increase in unprotected sex). The evidence comes from historical considerations. Consider the difference between the recent politically controversial HPV vaccine and the uneventful introduction of the HBV vaccine into the U.S. health system in the 1990s. These vaccines protect against the cancer-causing sexually transmitted diseases Human Papillomavirus and Hepatitis B, respectively. In the case of HBV, most people received their information about the vaccine and associated risks through their pediatrician, whereas many parents’ first exposure to information about HPV came through partisan news outlets. The reason the HPV vaccine received a political spotlight is because Merck, the manufacturer of the HPV vaccine Gardasil, attempted to get approval through a fast-track review process from the U.S. Food and Drug Administration and lobbied a nation-wide campaign directing state legislatures to add the vaccine to immunization schedules required for school enrollment. If successful, Merck would have positioned itself in a dominant market position against GlaxoSmithKline’s rival product, Cervarix. Without the fast track, both vaccines would have gone through the same process as the HBV vaccine, avoiding a political spotlight and receiving approval about three years later. Once in the political spotlight, however, the HPV vaccine lent itself to ideological objections. Some of these objections were religiously motivated, but not all (in fact, religious groups did not oppose the FDA approval of the HPV vaccine [60]).

In light of the above considerations, we investigate the socio-political characteristics to assess attitudes about vaccination. In particular, we consider how political ideology and trust affect vaccination beliefs for flu, pertussis (whooping cough), and measles. We select these diseases because of their contrastive features. Flu vaccination is chosen annually while vaccination for pertussis and measles are done during childhood, and measles outbreaks have received heightened media attention compared to flu and pertussis. We investigate two forms of trust: trust in government medical experts (such as Centers for Disease Control and Prevention) and trust in primary health care provider (such as pediatrician or family doctor). Furthermore, we investigate the relationship between trust and ideology, where ideology is conceived as a continuum ranging from very conservative, to moderate, to very liberal.

Our results, in brief, demonstrate that political ideology affects vaccine attitudes indirectly, by affecting a person’s trust in health-related information sources, and more directly as well. These findings are consistent with an earlier study by Rabinowitz et al. [61] One of the criticisms of this earlier study was that it made use of a convenience sample, rather than a nationally representative survey. Our study makes use of a larger and nationally representative sample. Thus our work constitutes an advance in knowledge of this topic by providing an important replication of earlier work done by others.

## Methods

### Data collection and sample characteristics

In order to test our hypotheses, we rely on data from a nationally representative online survey, collected from January 25–27, 2017. Our sample was provided by Survey Sampling International (SSI), a U.S.-based market research firm. After we obtained IRB exemption from our institution [Project Number: 17–007; exemption granted under category 2 at 45 CFR 46.101(b)(2)], SSI sent the link to our survey (which was programmed on Qualtrics) to 1,006 respondents. In this context, the survey firm ensured that our final sample would match known parameters of the U.S. adult population on five major dimensions: age, gender, income, ethnicity, and census region. This goal was achieved. As we show in S1 Table, our respondent pool approximates the overall citizenry of the U.S. very closely.

The survey consisted of three major sections. First, respondents were asked a number of questions about their political beliefs. Second, subjects answered survey items tapping into attitudes about vaccinations (described below). Finally, all participants provided information about basic demographic characteristics.

### Dependent variable

Our dependent variable taps into individual-level beliefs about vaccinations. We focused on three diseases: pertussis (whooping cough), measles, and influenza. Measuring vaccination attitudes is a non-trivial task. It seems likely that the overwhelming majority of our respondents received the vaccine for most preventable diseases at a very young age. As a result, asking respondents about their own immunization *record* would not necessarily capture their *beliefs* about the topic. An alternative approach would be to study the decisions that subjects make for their children. This too, is problematic since an exclusive focus on parents would decrease our sample size significantly and therefore compromise statistical power.

Our solution to this problem is as follows. We designed two *hypothetical* questions which correspond to slightly different scenarios. Question 1 simulates a low-risk setting. We asked our interviewees to imagine that they are currently “missing the vaccine for the following diseases but there is no immediate risk of getting infected.” Respondents then gave separate answers for pertussis, measles, and influenza and they indicated how likely/unlikely they would be to get vaccinated. Answer options were (1) very unlikely, (2) unlikely, (3) neither likely nor unlikely, (4) likely, (5) very likely, and (6) I don’t know. Question 2 corresponds to a high-risk scenario. Again, we asked respondents to imagine that they were missing the relevant vaccines. However, “now there is an outbreak of that disease in [their] community.” Here too, respondents gave separate answers for the three diseases of interest. The answer options remained the same.

Our approach leaves us with six analyzable variables (i.e., answers for three diseases in two different scenarios). We factor-analyzed these items and we expected to obtain two latent dimensions: one tapping into vaccination attitudes in high-risk scenarios and one capturing beliefs about immunizations in low-risk settings. This expectation is *not* supported. Our analysis reveals that all survey items clearly tap into one underlying dimension: the factor loadings for all variables are above 0.80. Furthermore, only one factor reaches an Eigenvalue of 1 or higher (Factor 1: 4.56). Given these findings, we created one latent construct (“Vaccination Attitudes”) and we used this item for our statistical analysis below. Higher values on this variable indicate more favorable views about vaccinations. Factor loadings for this latent construct are displayed in Table 1. A detailed breakdown of the associations between all vaccine-related variables can be found in S2 Table.

**Table 1:** Factor loadings.

Survey Item	Factor Loading
Vaccination Attitudes (Pertussis; Low Risk Scenario)	0.86
Vaccination Attitudes (Measles; Low Risk Scenario)	0.89
Vaccination Attitudes (Influenza; Low Risk Scenario)	0.83
Vaccination Attitudes (Pertussis; High Risk Scenario)	0.90
Vaccination Attitudes (Measles; High Risk Scenario)	0.90
Vaccination Attitudes (Influenza; High Risk Scenario)	0.85
Cronbach’s Alpha: 0.94 Eigenvalue of Estimated Factor: 4.56	

### Exogenous and mediator variables

Our main independent variable is political ideology. In order to capture this concept, we asked respondents to place themselves on a five-point scale ranging from “very liberal” to “very conservative.” About 9.9 percent of respondents self-identified as “very liberal”, 17.5 percent as “liberal”, 41.4 percent as “moderate”, 21.6 percent as “conservative”, and 9.6 percent as “very conservative.”

According to the theoretical framework discussed above, ideology should have a direct effect on vaccination attitudes. In addition, we also hypothesize that an individual’s political worldview should influence how much trust they place in their primary health care provider as well as government medical experts. According to Hypotheses 2 and 3, these two types of trust should then also affect vaccination attitudes. Expressed in more formal terms, we also expect an *indirect* effect of ideology on vaccination attitudes that is mediated by trust. We measured these two mediator variables by asking respondents to what extent they trust their family’s health care provider and government medical experts (such as Centers for Disease Control and Prevention) “regarding questions about health.” There were six response options: (1) strongly distrust, (2) somewhat distrust, (3) neither trust nor distrust, (4) somewhat trust, (5) strongly trust, and (6) I don’t know. All respondents who answered “I don’t know” were excluded from the analysis.

Finally, we introduce a standard set of control variables from the public opinion literature to account for other causes of our dependent variables: age, gender (male 1/0), education, income, and racial background (Caucasian 1/0). Correlations between all continuous variables in this paper can be found in Table 2. Descriptive Statistics for all variables can be found in S3 Table.

**Table 2:** Correlations between continuous variables.

	Age	Education	Income	Ideology	Trust (Gov. Medical Experts)	Trust (Health Care Provider)	Latent Vaccine Attitudes (DV)
Age							
Education	R = 0.07 (p<0.03)						
Income	R = 0.03 (p<0.35)	R = 0.43 (p<0.01)					
Ideology	R = 0.09 (p<0.01)	R = -0.05 (p<0.14)	R = 0.02 (p<0.58)				
Trust (Gov. Medical Experts)	R = -0.03 (p<0.48)	R = 0.05 (p<0.11)	R = 0.03 (p<0.30)	R = -0.18 (p<0.01)			
Trust (Health Care Provider)	R = 0.10 (p<0.01)	R = 0.05 (p<0.10)	R = 0.07 (p<0.02)	R = -0.01 (p<0.72)	R = 0.35 (p<0.01)		
Latent Vaccine Attitudes (DV)	R = -0.08 (p<0.02)	R = 0.14 (p<0.01)	R = 0.14 (p<0.01)	R = -0.17 (p<0.01)	R = 0.30 (p<0.01)	R = 0.29 (p<0.01)	

### Analytical approach

We use a structural equation model to test for both direct and indirect effects. Calculations were performed using STATA 14. It should be noted that the results of the model we present herein use as the dependent variable the latent construct, described above. However, to demonstrate the robustness of our results we also estimated six separate models using each of the six base constructs as the outcome variable. The results are substantively identical and can be found in S4 Table.

The following three indices (and standard cutoffs), recommended by Hu and Bentler [62], were used to evaluate the goodness of fit of the model: (a) the Standardized Root Mean Square Residual (SRMR), (b) the Root Mean Square Error of Approximation (RMSEA), and (c) the Comparative Fit Index (CFI); a model was considered to have a good fit if SRMR was below 0.05, RMSEA was below 0.05, and CFI was 0.95 or more. The results revealed that the model fits the data very well: SRMR = 0.02; RMSEA = 0.04; and CFI = 0.97.

## Results and discussion

### Direct effects

**Fig 1:**
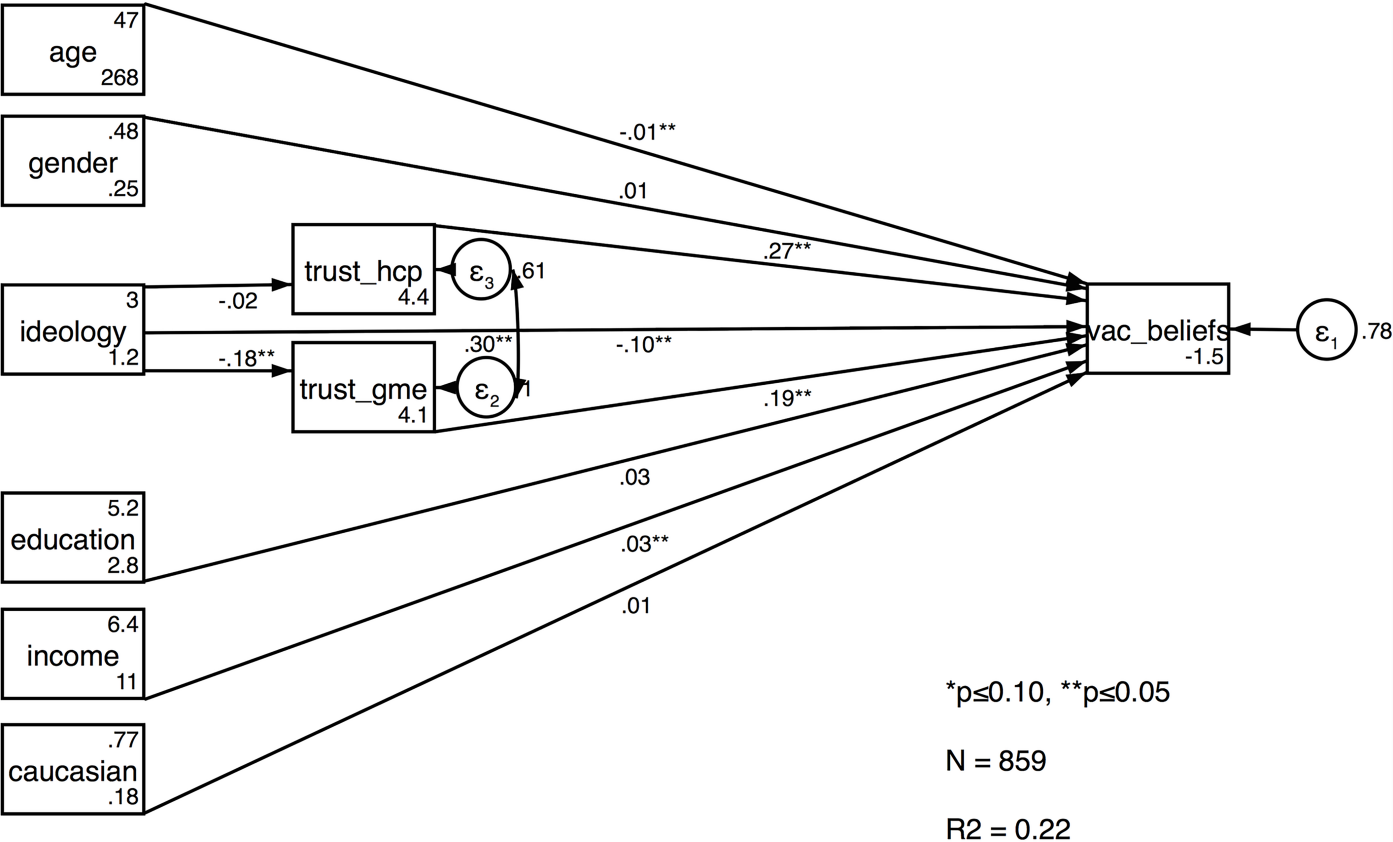
Path model results.

Fig 1 provides results from our path model. We report unstandardized coefficients, standard errors as well as p-values. As predicted by Hypothesis 1, ideology has a strong and statistically significant effect on vaccination attitudes (B = -0.10; std. error: 0.03; p<0.01). More specifically, conservative respondents are less likely to indicate that they would vaccinate against pertussis, measles, and influenza than other individuals. Furthermore, both trust in health care provider (B = 0.27; std. error: 0.04; p<0.01) and trust in government medical experts (B = 0.19; std. error: 0.03; p<0.01) have direct effects on our dependent variable. For both variables, the path coefficient is positive and statistically significant which suggests that people with faith in these two entities are also more likely to indicate that they would vaccinate if they missed the immunization. These empirical findings are in line with Hypotheses 2 and 3 of this paper.

### Indirect effects

According to Hypotheses 4 and 5, an individual’s political worldview should influence their level of trust in various health care-related information sources. Our statistical analysis provides evidence for only one of these paths. We see that ideology has a strong and statistically significant effect on trust in government medical experts (B = -0.18; std. error: 0.03; p<0.01). In particular, more conservative respondents tend to express lower levels of trust in institutions like the CDC than their less conservative counterparts. Contrary to our theoretical expectations however, we find no evidence in support of Hypothesis 5. In other words, an individual’s political worldview does not seem to influence the extent to which they trust their family’s primary health care provider (B = -0.02; std. error: 0.02; p<0.41).

These findings imply that there is, in fact, an indirect effect of ideology on vaccination attitudes that is mediated by trust in government medical experts. As we show in Table 3, the estimated size of this indirect effect is -0.04 (std. error: 0.01; p<0.01). This amounts to about 29 percent of the total ideology effect on our dependent variable (B = -0.14; std. error: 0.03; p<0.01). Taking into account all pathways in Fig 1, “strong conservatives” are thus estimated to score 0.56 points lower on our latent scale than “strong liberals.” This means that the overall effect of ideology is not only statistically significant but also substantively meaningful.

**Table 3:** Direct and indirect effects.

Outcome	Direct Effect	Indirect Effect	Total Effect
**Trust in Health Care Provider**			
Ideology → Trust in Health Care Provider	—	—	—
**Trust in Government Medical Experts**			
Ideology → Trust in Government Medical Experts	-0.18**	—	-0.18**
**Vaccination Attitudes (Latent Scale)**			
Trust in Health Care Provider → Vaccination Attitudes	0.27**	—	0.27**
Trust in Gov. Medical Experts → Vaccination Attitudes	0.19**	—	0.19**
Ideology → Vaccination Attitudes	-0.10**	-0.04**	-0.14**
Age → Vaccination Attitudes	-0.01**	—	-0.01**
Income → Vaccination Attitudes	0.03**	—	0.03**

*p≤0.10

**p≤0.05

### Discussion of control variables

Finally, we turn to a discussion of the direct effects of our control variables. Table 3 demonstrates that vaccination attitudes are not only a function of trust and ideology but also of other socio-demographic characteristics. In particular, age and income seem to affect how individuals think about vaccine choice. According to our results, older citizens have slightly more negative views about immunizations than younger respondents (B = -0.006; std. error: 0.002; p<0.01). By contrast, income (B = 0.03; std. error: 0.01; p<0.01) has a positive effect on this dependent variable. This suggests that vaccine attitudes are at least partially driven by the resources that respondents have at their disposal.

## Conclusion

Decisions regarding vaccination are more complicated than simply considering risks, costs, and benefits. In this paper we argued that socio-political characteristics of individuals shape their vaccination attitudes. More specifically, we examined the role of ideology, trust, and the relationship between these and attitudes about vaccination. Our findings corroborate analyses that show that the intent to vaccinate differs among conservatives and liberals with conservatives expressing less intent to vaccinate. Similarly, those with lower levels of trust in government medical experts are also less likely to express intent to vaccinate, and these individuals also tend to be conservative. What has been less understood, however, is the nature of the relationship between ideology and trust. Our findings suggest that ideology has two routes in affecting people’s vaccination attitude. One is direct, independent of trust. The other route goes through trust. That is, a person’s ideology impacts who they trust such that they can selectively credit information related to vaccine risks and benefits in ways that reflect their ideology. We thus establish a direction in the relationship between ideology and trust, namely from ideology to trust.

Our findings may provide insights into addressing growing vaccine refusal. Current strategies tend to be driven by a knowledge-deficit approach, attempting to persuade the public by appealing to risks. While we do see that vaccine attitudes are partially driven by resources, our findings suggest that the success of knowledge-deficit strategies will be limited by whether individuals trust the sources by which they are informed of risks and benefits, where this trust in turn can be limited by ideology. These results and conclusions are consistent with earlier work by Rabinowitz et al. [61]. There it is argued that in the domain of vaccination choice (in addition to other domains such as smoking, alcohol consumption, and sexual behavior), the perception of facts and beliefs, particularly perceptions of social norms, can differ between conservatives, moderates, and liberals. We add to this the importance of variation in trust across these ideologies. Thus, to better gauge expected success of vaccine campaigns, attention should be given to socio-political context, and where possible, measures should be taken to tailor messages appropriately.

## Supporting information

S1 Table Sample characteristics (compared to 2010 census). Our respondent pool approximates the overall citizenry of the U.S. very closely.
(DOCX)

S2 Table Correlation matrix–dependent variables. A detailed breakdown of the associations between all vaccine-related variables.
(DOCX)

S3 Table Descriptive statistics. Descriptive statistics for all variables.
(DOCX)

S4 Table Robustness checks. We additionally estimated six separate models using each of the six base constructs as the outcome variable. The results are substantively identical to those presented in the main text.
(DOCX)

S1 File Replication data. (DTA)

S2 File Replication commands. (TXT)
