# Supplementary material for: The influence of political ideology and trust on willingness to vaccinate
Source: PLoS One. 2018 Jan 25;13(1):e0191728. doi: 10.1371/journal.pone.0191728 (PMC5784985; doi:10.1371/journal.pone.0191728)
Supplement: S2 Table — A detailed breakdown of the associations between all vaccine-related variables. (DOCX) [file pone.0191728.s002.docx]

**Appendix Table S2. Correlation Matrix - Dependent Variables.**

|  | Likelihood of Vaccination (Pertussis; Low Risk Scenario) | Likelihood of Vaccination (Measles; Low Risk Scenario) | Likelihood of Vaccination (Influenza; Low Risk Scenario) | Likelihood of Vaccination (Pertussis; High Risk Scenario) | Likelihood of Vaccination (Measles; High Risk Scenario) | Likelihood of Vaccination (Influenza; High Risk Scenario) |
| --- | --- | --- | --- | --- | --- | --- |
| Likelihood of Vaccination (Pertussis; Low Risk Scenario) |  |  |  |  |  |  |
| Likelihood of Vaccination (Measles; Low Risk Scenario) | R= 0.89  (p<0.01) |  |  |  |  |  |
| Likelihood of Vaccination (Influenza; Low Risk Scenario) | R= 0.70  (p<0.01) | R= 0.73  (p<0.01) |  |  |  |  |
| Likelihood of Vaccination (Pertussis; High Risk Scenario) | R= 0.75  (p<0.01) | R= 0.72  (p<0.01) | R= 0.64  (p<0.01) |  |  |  |
| Likelihood of Vaccination (Measles; High Risk Scenario) | R= 0.70  (p<0.01) | R= 0.78  (p<0.01) | R= 0.64  (p<0.01) | R= 0.90  (p<0.01) |  |  |
| Likelihood of Vaccination (Influenza; High Risk Scenario) | R= 0.59  (p<0.01) | R= 0.61  (p<0.01) | R= 0.83  (p<0.01) | R= 0.77  (p<0.01) | R= 0.78  (p<0.01) |  |
