# Supplementary material for: The influence of political ideology and trust on willingness to vaccinate
Source: PLoS One. 2018 Jan 25;13(1):e0191728. doi: 10.1371/journal.pone.0191728 (PMC5784985; doi:10.1371/journal.pone.0191728)
Supplement: S3 Table — Descriptive statistics for all variables. (DOCX) [file pone.0191728.s003.docx]

**Appendix Table S3. Descriptive Statistics.**

| **Variable** | **Min** | **Max** | **Mean** | **Std. Dev.** | **Number of responses** | **Missing (incl. dk)** |
| --- | --- | --- | --- | --- | --- | --- |
| *Dependent Variable* |  |  |  |  |  |  |
| Vaccination Attitudes (latent variable) | -2.20 | 1.03 | 0.00 | 0.99 | 940 | 56 |
|  |  |  |  |  |  |  |
| *Mediator Variables* |  |  |  |  |  |  |
| Trust in Primary Health Care Provider | 1 | 5 | 5.30 | 0.80 | 989 | 17 |
| Trust in Government Medical Experts | 1 | 5 | 3.57 | 1.04 | 989 | 17 |
|  |  |  |  |  |  |  |
| *Exogenous Variables* |  |  |  |  |  |  |
| Ideology | 1 | 5 | 3.03 | 1.08 | 945 | 61 |
| Age | 18 | 97 | 46.5 | 16.3 | 997 | 9 |
| Gender (Male = 1/0) | 0 | 1 | 0.47 | 0.49 | 1,001 | 5 |
| Education | 1 | 8 | 5.02 | 1.69 | 1,005 | 1 |
| Income | 1 | 12 | 6.17 | 3.41 | 1,005 | 1 |
| Race (Caucasian 1/0) | 0 | 1 | 0.75 | 0.43 | 1,006 | 0 |
