# Supplementary material for: The influence of political ideology and trust on willingness to vaccinate
Source: PLoS One. 2018 Jan 25;13(1):e0191728. doi: 10.1371/journal.pone.0191728 (PMC5784985; doi:10.1371/journal.pone.0191728)
Supplement: S4 Table — We additionally estimated six separate models using each of the six base constructs as the outcome variable. The results are substantively identical to those presented in the main text. (DOCX) [file pone.0191728.s004.docx]

**Appendix Table S4. Robustness Checks**

| Path | Direct Effect | Indirect Effect | Total Effect |
| --- | --- | --- | --- |
| **Model S1: Dependent Variable: Vaccination Attitudes; Pertussis; Low Risk Scenario** | | | |
| Ideology 🡺 Trust in Health Care Provider | -- | -- | -- |
| Ideology 🡺 Trust in Government Medical Experts | -0.18** | -- | -0.18** |
| Trust in Health Care Provider 🡺  Vaccination Attitudes | 0.26** | -- | 0.26** |
| Trust in Gov. Medical Experts 🡺  Vaccination Attitudes | 0.28** | -- | 0.28** |
| Ideology 🡺 Vaccination Attitudes | -0.12** | -0.05** | -0.17** |
|  |  |  |  |
| **Model S2: Dependent Variable: Vaccination Attitudes; Measles; Low Risk Scenario** | | | |
| Ideology 🡺 Trust in Health Care Provider | -- | -- | -- |
| Ideology 🡺 Trust in Government Medical Experts | -0.18** | -- | -0.18** |
| Trust in Health Care Provider 🡺  Vaccination Attitudes | 0.28** | -- | 0.28** |
| Trust in Gov. Medical Experts 🡺  Vaccination Attitudes | 0.23** | -- | 0.23** |
| Ideology 🡺 Vaccination Attitudes | -0.15** | -0.05** | -0.20** |
|  |  |  |  |
| **Model S3: Dependent Variable: Vaccination Attitudes; Influenza; Low Risk Scenario** | | | |
| Ideology 🡺 Trust in Health Care Provider | -- | -- | -- |
| Ideology 🡺 Trust in Government Medical Experts | -0.18** | -- | -0.18** |
| Trust in Health Care Provider 🡺  Vaccination Attitudes | 0.34** | -- | 0.34** |
| Trust in Gov. Medical Experts 🡺  Vaccination Attitudes | 0.25** | -- | 0.25** |
| Ideology 🡺 Vaccination Attitudes | -0.08* | -0.05** | -0.13** |
|  |  |  |  |
| **Model S4: Dependent Variable: Vaccination Attitudes; Pertussis; High Risk Scenario** | | | |
| Ideology 🡺 Trust in Health Care Provider | -- | -- | -- |
| Ideology 🡺 Trust in Government Medical Experts | -0.17** | -- | -0.17** |
| Trust in Health Care Provider 🡺  Vaccination Attitudes | 0.36** | -- | 0.36** |
| Trust in Gov. Medical Experts 🡺  Vaccination Attitudes | 0.23** | -- | 0.23** |
| Ideology 🡺 Vaccination Attitudes | -0.11** | -0.04** | -0.15** |
|  |  |  |  |
| **Model S5: Dependent Variable: Vaccination Attitudes; Measles; High Risk Scenario** | | | |
| Ideology 🡺 Trust in Health Care Provider | -- | -- | -- |
| Ideology 🡺 Trust in Government Medical Experts | -0.18** | -- | -0.18** |
| Trust in Health Care Provider 🡺  Vaccination Attitudes | 0.31** | -- | 0.31** |
| Trust in Gov. Medical Experts 🡺  Vaccination Attitudes | 0.21** | -- | 0.21** |
| Ideology 🡺 Vaccination Attitudes | -0.13** | -0.04** | -0.17** |
|  |  |  |  |
| **Model S6: Dependent Variable: Vaccination Attitudes; Influenza; High Risk Scenario** | | | |
| Ideology 🡺 Trust in Health Care Provider | -- | -- | -- |
| Ideology 🡺 Trust in Government Medical Experts | -0.18** | -- | -0.18** |
| Trust in Health Care Provider 🡺  Vaccination Attitudes | 0.36** | -- | 0.36** |
| Trust in Gov. Medical Experts 🡺  Vaccination Attitudes | 0.21** | -- | 0.21** |
| Ideology 🡺 Vaccination Attitudes | -0.10** | -0.04** | -0.14** |
| *p≤0.10, **p≤0.05 |  |  |  |
